# Hospitalizations of cancer patients in the last month of life: quality indicator scores reveal large variation between four European countries in a mortality follow-back study

**DOI:** 10.1186/1472-684X-13-54

**Published:** 2014-11-27

**Authors:** Maaike L De Roo, Anneke L Francke, Lieve Van den Block, Gé A Donker, Jose E Lozano Alonso, Guido Miccinesi, Sarah Moreels, Bregje D Onwuteaka-Philipsen, Andrea Salvetti, Luc Deliens, on behalf of EURO IMPACT

**Affiliations:** Department of Public and Occupational Health, Expertise Center of Palliative Care, VU University Medical Center, EMGO Institute for Health and Care Research, Van der Boechorststraat 7, 1081 BT Amsterdam, the Netherlands; NIVEL, Netherlands Institute for Health Services Research, P.O. Box 1568, 3500 BN Utrecht, the Netherlands; Vrije Universiteit Brussel (VUB), End-of-life Care Research Group Vrije Universiteit Brussel (VUB) and Ghent University, Laarbeeklaan 103, 1090 Brussels, Belgium; Public Health Directorate General, Regional Ministry of Health, Government of Castilla y León, Paseo de Zorrilla 1, 47071 Valladolid, Spain; Clinical and Descriptive Epidemiology Unit, Cancer Prevention and Research Institute, ISPO, via Oblate 2, Pal 28/A, 50141 Florence, Italy; Scientific Institute of Public Health, Public Health and Surveillance, Health Services Research, Juliette Wytsmanstraat 14, 1050 Brussels, Belgium; Italian Society of General Practioners (SIMG), Via del Pignoncino 9-11, 50142 Florence, Italy

**Keywords:** Hospitalizations, Palliative care, Quality indicators

## Abstract

**Background:**

Repeated and long hospitalizations of cancer patients at the end of life have been suggested as indicators of low quality of palliative care. Comparing the care delivered between different countries with the help of these quality indicators may identify opportunities to improve practice. Our objective is twofold: firstly, to describe the scores for the existing quality indicators “the percentage of time spent in hospital” and “the proportion of adult patients with more than one hospitalization in the last 30 days of life” in populations of cancer patients in four European countries and to see whether these countries met previously defined performance standards; secondly, to assess whether these scores are related to receiving palliative care from their GP.

**Methods:**

A mortality follow-back study was conducted, based on data recorded by representative GP networks for samples of cancer patients living at home who died non-suddenly in Belgium (n = 500), the Netherlands (n = 310), Italy (n = 764), and Spain (n = 224).

**Results:**

The quality indicator score for “the percentage of time spent in hospital” in the last month of life was 14.1% in the Netherlands, 17.7% in Spain, 22.2% in Italy, and 24.6% in Belgium, which means that none of the countries met the performance standard of <10%. For the “proportion of patients with more than one hospitalization in the last 30 days of life”, two countries met the performance standard of <4%: the Netherlands (0.6%) and Italy (3.1%). Spain had a score of 4.0% and Belgium scored 5.4%. When patients received palliative care from their GP, significantly less time was spent in hospital in the last month and fewer hospitalizations took place.

**Conclusions:**

European countries differ regarding the frequency and duration of hospitalizations of cancer patients in the last month of life. This reflects country-specific differences in the organization of palliative care and highlights the important role of the GP in palliative care provision.

## Background

High rates of hospitalization at the end of life may be an indication that palliative care is of suboptimal quality, since these hospitalizations can be associated with offering aggressive and futile treatments [1, 2], with too much focus on life prolongation rather than the patient’s quality of life and the relief of symptom burden, with inadequate communication about the patient’s care preferences or with the limited availability or use of palliative home-care services [3]. Although some hospitalizations may be inevitable [4, 5], there may be potential to reduce the number and duration of hospitalizations [6], e.g. by providing appropriate support from general practitioners [1, 7].

Long or repeated hospital admissions at the end of life have been suggested as indicators that palliative care is of a poor quality [1, 8, 9]. Several quality indicators for palliative care concerning the frequency and duration of hospitalizations at the end of life have already been developed [1, 9–12]. Measuring these quality indicators can give insights into areas where the quality of care is not optimal, subsequently enabling priorities to be set for quality improvement [12]. In this study, we used two quality indicators regarding hospitalizations, selected specifically because they could be derived from the existing data records of general practitioners (GPs) in Belgium, the Netherlands, Italy, and Spain. The first is “the percentage of time spent in hospital”, coming from a set of quality indicators developed in Italy for palliative home care [9]. The second quality indicator used concerns “the proportion with more than one hospitalization in the last 30 days of life”. This quality indicator is part of an indicator set that was developed in the United States for cancer patients [1, 10, 11]. These indicators have specific performance standards: namely that less than 4% of cancer patients should have more than one hospitalization in the last month of life [10] and that less than 10% of time should be spent in hospital [9]. Using these existing indicators, instead of constantly developing new indicators for palliative care offers advantages. In this case, deriving these indicators from data collected by existing registrations by GPs, we further tested the usefulness of these indicators in international comparative research.

Comparing the care delivered between different countries may help identify opportunities to improve practice [13], particularly when the comparison includes an investigation of the factors that are associated with poor or better quality indicator scores. In this paper, we therefore also look at whether there is a relationship with the delivery of palliative care by GPs. Previous studies have shown that the provision of palliative care by GPs is associated with less time spent in hospital and fewer hospitalizations [4, 5]. It is also important to examine whether there is a relationship with GP provision of palliative care because the roles of GPs differ between countries. In some countries, like the Netherlands and Spain, GPs function as gatekeepers [14] to hospital care: except in very acute situations, patients need a formal referral from the GP to see a medical specialist in a hospital. Hence, this provides an opportunity for preventing avoidable hospitalizations. Although GPs in Belgium and Italy do not have this strict gatekeeper function, they are still central professionals in the healthcare system and have a coordinating role, since most people have their “own” GP whom they consult when they have medical problems [15]. Another aspect of the GP’s function that differs between countries is their role in the provision of palliative care. In the Netherlands, the GP plays a central role in the delivery of generalist palliative care at home [16–18]. In the other three countries, the GP shares the responsibility of palliative care delivery with generalist or specialist palliative-care home teams [14, 15, 18–20].

This paper addresses the following research questions:

1. What is a) the percentage of time spent in hospital in the last month of life, and b) the proportion of cancer patients with more than one hospitalization in the last 30 days of life who lived at home and who died non-suddenly in Belgium, the Netherlands, Italy, or Spain?
2. Do the countries meet the performance standards defined for these two quality indicators?
3. Do these quality indicator scores differ between the cancer patients who received palliative care from their general practitioner and those who did not receive palliative care from their GP?

## Methods

### Study design

This paper is based on data from the European Sentinel GP Networks Monitoring End-of-Life Care (EURO SENTI-MELC) study, a mortality follow-back study on monitoring end-of-life care in Belgium, the Netherlands, Spain, and Italy. For this study, we used data from the nationally representative GP networks [14] collected in 2009 (all countries except Spain), 2010 (all four countries) and 2011 (Spain only). The GP sentinel networks cover 1.8% and 0.8% of the Belgian and Dutch national populations respectively [14, 21, 22]. In Spain, the two sentinel networks involved in this study account for 3.5% of the patient population in the Castilla y León region (in the northwest) and 2.2% in the Comunitat Valenciana region (in the east) [14, 23]. The Italian data came from a new GP network set up for this study [24] and were collected from nine of the 146 health districts, covering about 4% of the national patient population [14]. The participating GPs in all four countries were representative for the general population of GPs in each country (or health districts in Italy and regions in Spain) in terms of age, gender, and geographical distribution [14, 25, 26].

### Study population

Since one of the two quality indicators selected was developed for a cancer population and the other for a population receiving home care, we decided to focus on a population of cancer patients who lived at home in the last month of life. The data were analyzed of deceased adult cancer patients (aged 18 and above), who had died non-suddenly according to their GP. Since this study examines the care delivered at the end of life, the data of people who died suddenly and unexpectedly according to their GP were excluded, leaving a population that was eligible for palliative care [21].

### Data collection

In the EURO SENTI-MELC study, GPs recorded the characteristics of recently deceased patients on a weekly basis using a standardized questionnaire. Recall bias was minimized by requiring data entry to be no more than one week after the GP had been informed of the patient’s death [14]. In the questionnaire, GPs were asked about the place of death and place(s) of residence in the last three months before death, as well as the length of stay in specific care settings in the last 30 days before death. Thus, the number of hospitalizations and the length of stay in hospital in the last month of life could be deduced. GPs were asked to indicate whether they provided palliative care by the following question: “Did you provide palliative care to this patient?” [“no”; “yes, but not until death”; “yes, until death” (dichotomized into “yes” and “no”)].

### Informed consent and patient anonymity

After being informed of the objectives and procedures of the study, participating GPs gave written informed consent at the beginning of each registration year. Strict procedures regarding patient anonymity were employed during data collection and entry; every patient was assigned an anonymous reference code by their GP and any identifying patient and GP data (such as date of birth, postcode, and GP identification number) were replaced with aggregate categories or anonymous codes.

### Ethical approval

The protocol of this study was approved by the Ethical Review Board of Brussels University Hospital of the Vrije Universiteit Brussel (2004), Belgium, and the Local Ethical Committee, ‘Comitato Etico della Azienda U.S.L. n. 9 di Grosseto’ (2008), Tuscany, Italy. In the Netherlands and Spain, no ethical approval is required for the posthumous collection of anonymous patient data.

### Statistical analysis

The quality indicator “the percentage of time spent in hospital” is calculated using “number of days in hospital during home palliative care” as the numerator and “the total number of days of home palliative care” as the denominator. The performance standard “less than 10% of time should be spent in hospital” [9] was originally specified for patients who received home palliative care. In this study, it is calculated for the last month of life, for cancer patients regardless of whether they received home palliative care. The second quality indicator, “the proportion with more than one hospitalization in the last 30 days of life”, was calculated using “the number of patients who died from cancer and had more than one hospitalization in the last 30 days of life” as the numerator and “the number of patients who died from cancer” as the denominator. We used the original performance standard: “less than 4% of cancer patients should have more than one hospitalization in the last month of life” [10].

To enable a valid comparison between countries, the quality indicator scores were standardized for patients’ gender, age at death, and cancer type, using the distribution observed in the study population as a whole as the reference distribution.

To test whether these quality indicator scores differed significantly between the patients who received palliative care from their GP and those who did not, we used a Mann Whitney U test for “the percentage of time spent in hospital” and a Fisher’s Exact test for “the proportion with more than one hospitalization in the last 30 days of life”. Standardization of the quality indicator scores to enable valid comparison between the two groups was not applied, since the two groups did not differ significantly in terms of gender, age at death, and cancer type. The analyses were performed using IBM SPSS Statistics software, Version 20.0 (IBM Corp., 2011, Armonk, NY), with significance level α <0.05.

## Results

### Description of the sample

**Figure 1:**
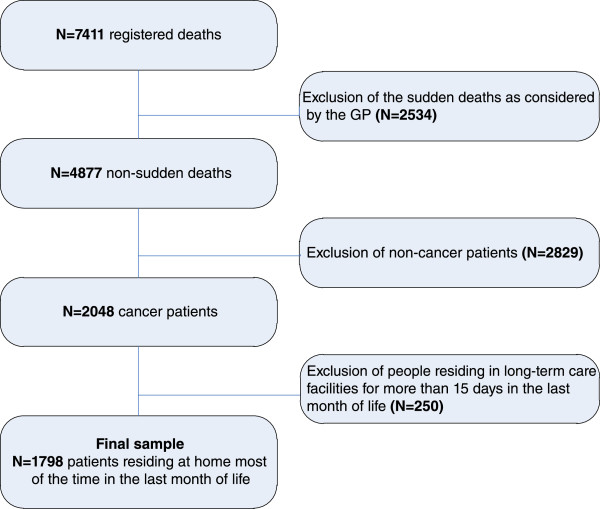
**Flowchart of the sample selection.**

The total sample in this study consisted of 1798 patients: 500 for Belgium, 310 for the Netherlands, 764 for Italy and 224 for Spain (see Figure 1). In all countries, the majority of the patients in the samples were male. About one fifth of the Italian and Spanish samples were aged 85 or older, whereas this group of the very elderly was smaller in Belgium (13.9%) and the Netherlands (11.3%) (Table 1). Lung cancer and colorectal cancer were the most common types of cancer in all four countries (Table 1). The proportion of cancer patients in each country receiving palliative care from their GP ranged from 61.4% (Belgium) to 73.9% (the Netherlands) (Table 1).

**Table 1:** **Characteristics of study population per country (N = 1798)**

	Belgium (N = 500) N (%)	The Netherlands (N = 310) N (%)	Italy (N = 764) N (%)	Spain (N = 224) N (%)
Gender*				
Female	210 (42.0)	138 (44.8)	344 (45.0)	69 (31.1)
Male	290 (58.0)	170 (55.2)	420 (55.0)	153 (68.9)
**Age at death** ^**†**^				
18-64 years	150 (30.3)	88 (28.4)	180 (23.6)	51 (22.8)
65-84 years	276 (55.8)	187 (60.3)	430 (56.3)	132 (58.9)
85 years and older	69 (13.9)	35 (11.3)	154 (20.2)	41 (18.3)
**Cancer type** ^**‡**^				
Lung cancer	135 (27.0)	78 (26.4)	174 (27.6)	44 (20.2)
Breast cancer	39 (7.8)	30 (10.2)	51 (8.1)	11 (5.0)
Colorectal cancer	59 (11.8)	34 (11.5)	92 (14.6)	42 (19.3)
Prostate cancer	20 (4.0)	22 (7.5)	30 (4.8)	22 (10.1)
Other	247 (49.4)	131 (44.4)	283 (44.9)	99 (45.4)
**GP provided palliative care** ^**§**^				
No	193 (38.6)	79 (26.1)	277 (36.3)	62 (28.8)
Yes	307 (61.4)	224 (73.9)	486 (63.7)	153 (71.2)

*Missing values: Belgium no missing values, the Netherlands N = 2, Italy no missing values, Spain N = 2.

^†^Missing values: Belgium N = 5, the Netherlands, Italy and Spain no missing values.

^‡^Missing values: Belgium no missing values, the Netherlands N = 15, Italy N = 134, Spain N = 6.

^§^Missing values: Belgium no missing values, the Netherlands N = 7, Italy N = 1, Spain N = 9.

Overall, GPs knew where the patient was residing in the last 30 days of life in 96% of the cases. The percentage of GPs who did not know where the patient resided was lowest in the Netherlands (1%), and highest in Spain (14%). GPs in Belgium and Italy did not know where the patient resided in the last month of life for 3% of their patients (not shown in Tables).

### Quality indicator “the percentage of time spent in hospital”

The Netherlands had the lowest percentage of time spent in hospital in the last month of life (14.1%), and Belgium the highest percentage (24.6%) (Table 2). If we compare quality indicator scores between the cancer patient group who did receive GP palliative care and the group who did not, we see that in all countries the quality indicator scores are significantly lower, i.e. less time was spent in hospital, for the group that received GP palliative care (Table 2). Only the group of cancer patients who received palliative care from their GP in the Netherlands met the performance standard of 10%, as they spent only 7.5% of their last month in hospital.

**Table 2:** **Quality indicator scores per country and comparing cancer patients who received palliative care from their GP and those who did not**

	“Percentage of time spent in hospital” in the last month of life (%)				“Proportion with more than one hospitalization in the last 30 days of life” (%)			
Performance Standard* ^,†^	<10%				<4%			
	Belgium	The Netherlands	Italy	Spain	Belgium	The Netherlands	Italy	Spain
**Total population per country** ^**‡**^	24.6	14.1	22.2	17.7	5.4	0.6	3.1	4.0
**Did not receive palliative care from the GP** ^**§**^	39.7^**||**^	34.7^**||**^	29.3^**||**^	32.3^**||**^	8.3	1.3	5.1^**¶**^	8.1
**Received palliative care** **from the GP** ^**§**^	16.1^**||**^	7.5^**||**^	18.7^**||**^	11.8^**||**^	4.2	0.4	2.1^**¶**^	3.3

*Performance standard for the quality indicator “the percentage of time spent in hospital” in the last month of life is 10% [9].

^†^Performance standard for the for the quality indicator “the percentage of patients who had more than 1 hospitalization in the last month of life” is 4% [1, 10, 11].

^‡^These percentages are standardized for gender, age and cancer type.

^§^These percentages are not standardized for gender, age and cancer type, since these characteristics did not differ significantly between the two groups in each country.

^||^Mann Whitney U test showed significant difference, p < 0.001.

^¶^Fisher’s Exact test showed significant difference, p < 0.05.

### Quality indicator “the proportion with more than one hospitalization in the last 30 days of life”

The Netherlands had the lowest proportion with more than one hospitalization in the final month of life (0.6%), followed by Italy (3.1%). Spain (4.0%) and Belgium (5.4%) had a higher proportion of multiple hospitalizations (Table 2). The performance standard of less than 4% was thus met in two countries: the Netherlands and Italy (Table 2).

There were fewer rehospitalizations among the group of cancer patients who received GP palliative care, although a significant difference was only found in Italy (Table 2). The performance standard of 4% was met for the patients receiving GP palliative care in three countries: the Netherlands (0.4%), Italy (2.1%), and Spain (3.3%). The Belgian score of 4.2% almost met the performance standard. In the Netherlands, the performance standard was also met for the group of patients who did not receive palliative care from their GP (Table 2).

## Discussion

The percentage of time spent in hospital during the last month of life varied between the four countries, ranging from 14.1% (the Netherlands) to 24.6% (Belgium), while the proportion of patients with more than one hospitalization ranged from 0.6% (the Netherlands) to 5.4% (Belgium). The group of patients who received palliative care from their GP spent significantly less time in hospital and had fewer hospitalizations in the last month of life.

The original studies presenting these quality indicators [1, 9–11] also specified a performance standard. For the indicator concerning the time spent in hospital in the last month of life, none of the four countries met the performance standard (i.e. less than 10% of time should be spent in hospital) in our study. One could argue that we did not evaluate the performance of home palliative care, as was the case in the original study in Italy [9] and therefore cannot apply this this performance standard to our data, because while patients were living at home in our study, they were not necessarily receiving home palliative care. Nevertheless, even when we calculated the quality indicator scores for the patients who received palliative care from their GP, only the Netherlands (7.5%) met this performance standard. This could raise the question of whether a new performance standard needs to be defined when measuring this quality indicator nationwide. In this case, an alternative could be to apply the “best-practice norm” principle: take the best-scoring country’s score as the target other countries should aim for in the future.

For the other indicator, concerning the percentage of cancer patients who were hospitalized more than once in the last month of life, the performance standard (i.e. less than 4% of cancer patients should have more than one hospitalization in the last month of life) was not achieved in two of the four countries in our study (i.e. Spain and Belgium, with 4.0% and 5.4% of patients respectively having more than one hospitalization in the last month). This suggests this performance standard is a feasible goal and can be used as such in the future. The performance standard could even be updated following repeated measurements of these quality indicators, resulting in continuous quality improvement [10].

The between-country differences in quality indicator scores found in this study may reflect differences between these countries in the organization of palliative care. One of these differences may be the role of the GP in the provision of health care in general, and especially in the provision of palliative care. The high degree of responsibility assigned to GPs in the Netherlands, both as general gatekeepers [14] and specifically in the delivery of palliative care [16, 17], could be a reason for the fact that hospitalizations in the Netherlands are shorter and rehospitalizations are less frequent. Spain and Belgium have comparable rates, suggesting that the general gatekeeper function of the GP in Spain [14] may not have as much effect on hospitalizations as the fact that the organization of palliative care is the shared responsibility of both GPs and palliative home-care teams [27]. The latter is also the case in Belgium [15]. Despite the fact that in Italy palliative home care is mainly provided by multidisciplinary home teams [18, 19], the percentage of time spent in hospital in Italy is relatively high: 22.2%. Another study following an Italian cohort and US cohort in the year after the diagnosis of cancer revealed that the number of hospital admissions was the same in both countries but the mean number of days spent in hospital in Italy was double that of the US cohort [13]. Two potential causes were suggested: the fact that in Italy patients also stay in hospital for e.g. pre-intervention diagnostic tests, whereas in the US these tests were performed in an out-patient setting; and the fact that hospice programs in the US are more established than in Italy, possibly resulting in a higher number of hospitalizations for end-of-life care in Italy [13].

This is in line with the important finding of this study that among the group of patients where the GP provided palliative care, less time was spent in hospital in the last month of life, and multiple hospitalizations were less frequent. We cannot provide insight into the causality in this association due to the design of the study. It might be that patients could stay at home because they insisted on staying at home, had an informal caregiver at home, or had a low symptom burden, and therefore were in the right place to get palliative care from their GP. Nevertheless, this finding highlights the importance of the GP in the organization of palliative care, and the challenge for the GP and home-care services to reduce the number of potentially avoidable hospitalizations.

### Strengths and limitations

This is the first cross-national study using existing data to compare the length and number of hospitalizations in the last month of life, and to assess their function as quality indicators. A strength of the study is that it seems feasible to calculate the scores of these two quality indicators based on data gathered by GPs, as GPs knew where the patient was residing in the last 30 days of life in 96% of the non-sudden cancer deaths. Consequently, existing GP networks are a feasible candidate for a continuous monitor of some aspects of the quality of palliative care. Nevertheless, there are limitations when using GP networks to collect data. We cannot fully exclude the inaccurate judgment by GPs of patient deaths as being sudden and unexpected.

There may be a bias as GPs may not have been informed or aware of all transfers of the patient to and from hospital or they missed some transitions in the course of recording the data. Due to the anonymous coding of the data collected in the GP networks, we could not validate this information with hospital registries or insurance data. To minimize recall bias, GPs reported on a weekly basis.

Furthermore we do not have information on the reason for hospitalizations in the last month, because we used data recorded by existing GP networks, which did not contain information on this subject. For the same reason, we cannot provide information about whether these hospitalizations were elective or via the emergency department, nor whether they were potentially avoidable or unavoidable. In addition, the availability of hospices and palliative care units might influence whether patients are hospitalized or not in the last month of life. The existing registrations used in this study did not provide any data on the availability of hospices and palliative care units and whether patients with uncontrolled symptoms may have no choice but to be hospitalized. Further research could examine these issues more in-depth.

Another limitation is that GPs themselves stated whether they had provided palliative care and we could not examine the validity of this self-reported palliative care provision. We have no detailed information on what GPs considered as “providing palliative care” and were therefore unable to verify whether these definitions were consistent with existing expert definitions. Some GPs may consider care for patients with chronic diseases as palliative care, whereas other consider this as regular GP care. Therefore this study reflects the delivery of what GPs themselves perceive to be palliative care. However, as our study is limited to deceased *cancer* patients, inter-doctor variation is less likely than would be the case in a study of all deceased patients.

## Conclusion

“The percentage of time spent in hospital” in the last month of life and “the proportion with more than one hospitalization in the last 30 days of life” are quality indicators that can be collected with the use of existing sentinel networks of GPs. Quality indicator scores reveal substantial differences between countries, reflecting country-specific differences in the organization of palliative care. In the group of patients who received palliative care from their GP, there were fewer hospitalizations and significantly less time was spent in hospital in the last month, highlighting the important role of the GP in palliative care provision.

## Authors’ information

EURO IMPACT collaborators: Van den Block Lieve, De Groote Zeger, Brearley Sarah, Caraceni Augusto, Cohen Joachim, Francke Anneke, Harding Richard, Higginson Irene J, Kaasa Stein, Linden Karen, Miccinesi Guido, Onwuteaka-Philipsen Bregje, Pardon Koen, Pasman Roeline, Pautex Sophie, Payne Sheila, Deliens Luc.

## References

[1] Earle CC, Park ER, Lai B, Weeks JC, Ayanian JZ, Block S (2003). Identifying potential indicators of the quality of end-of-life cancer care from administrative data. J Clin Oncol.

[2] Mezey M, Dubler NN, Mitty E, Brody AA (2002). What impact do setting and transitions have on the quality of life at the end of life and the quality of the dying process?. Gerontologist.

[3] Earle CC, Landrum MB, Souza JM, Neville BA, Weeks JC, Ayanian JZ (2008). Aggressiveness of cancer care near the end of life: is it a quality-of-care issue?. J Clin Oncol.

[4] Van den Block L, Deschepper R, Drieskens K, Bauwens S, Bilsen J, Bossuyt N, Deliens L (2007). Hospitalisations at the end of life: using a sentinel surveillance network to study hospital use and associated patient, disease and healthcare factors. BMC Health Serv Res.

[5] Abarshi E, Echteld M, Van den Block L, Donker G, Deliens L, Onwuteaka-Philipsen B (2010). Transitions between care settings at the end of life in the Netherlands: results from a nationwide study. Palliat Med.

[6] Gott M, Frey R, Robinson J, Boyd M, O’Callaghan A, Richards N, Snow B (2013). The nature of, and reasons for, ‘inappropriate’ hospitalisations among patients with palliative care needs: A qualitative exploration of the views of generalist palliative care providers. Palliat Med.

[7] Hjermstad MJ, Kolflaath J, Lokken AO, Hanssen SB, Normann AP, Aass N (2013). Are emergency admissions in palliative cancer care always necessary? Results from a descriptive study. BMJ Open.

[8] Barbera L, Paszat L, Qiu F (2008). End-of-life care in lung cancer patients in Ontario: aggressiveness of care in the population and a description of hospital admissions. J Pain Symptom Manage.

[9] Peruselli C, Marinari M, Brivio B, Castagnini G, Cavana M, Centrone G, Magni C, Merlini M, Scaccabarozzi GL, Paci E (1997). Evaluating a home palliative care service: development of indicators for a continuous quality improvement program. J Palliat Care.

[10] Earle CC, Neville BA, Landrum MB, Souza JM, Weeks JC, Block SD, Grunfeld E, Ayanian JZ (2005). Evaluating claims-based indicators of the intensity of end-of-life cancer care. Int J Qual Health Care.

[11] National Quality Forum (2006). National Voluntary Consensus Standards for Symptom Management and End-of-Life Care in Cancer Patients.

[12] De Roo ML, Leemans K, Claessen SJJ, Cohen J, Pasman HR, Deliens L, Francke AL (2013). Quality indicators for palliative care: update of a systematic review. J Pain Symptom Manage.

[13] Gigli A, Warren JL, Yabroff KR, Francisci S, Stedman M, Guzzinati S, Giusti F, Miccinesi G, Crocetti E, Angiolini C, Mariotto A (2013). Initial treatment for newly diagnosed elderly colorectal cancer patients: patterns of care in Italy and the United States. J Natl Cancer Inst Monogr.

[14] Van den Block L, Onwuteaka-Philipsen B, Meeussen K, Donker G, Giusti F, Miccinesi G, Van Casteren V, Alonso T, Zurriaga O, Deliens L (2013). Nationwide continuous monitoring of end-of-life care via representative networks of general practitioners in Europe. BMC Fam Pract.

[15] Desmedt M, Michel H (2002). Palliative home care: improving co-operation between the specialist team and the family doctor. Support Care Cancer.

[16] Ministerie van Volksgezondheid WeS: *Verankering van Palliatieve Zorg In De Praktijk [Embedding of Palliative Care in Practice]*. [http://www.rijksoverheid.nl/documenten-en-publicaties/kamerstukken/2011/01/14/verankering-van-palliatieve-zorg-in-de-praktijk.html]

[17] Council of Europe: *Recommendation Rec (2003) 24 of the Committee of Ministers to member states on the organisation of palliative care*. [http://www.coe.int/T/DG3/Health/Source/Rec(2003)24_en.pdf]

[18] Sbanotto A, Burnhill R (1998). Palliative care in Italy: the current situation. Support Care Cancer.

[19] Costantini M, Toscani F, Gallucci M, Brunelli C, Miccinesi G, Tamburini M, Paci E, Di Giulio P, Peruselli C, Higginson I, Addington-Hall J (1999). Terminal cancer patients and timing of referral to palliative care: a multicenter prospective cohort study. Italian cooperative research group on palliative medicine. J Pain Symptom Manage.

[20] Abarshi E, Echteld MA, Van den Block L, Donker G, Bossuyt N, Meeussen K, Bilsen J, Onwuteaka-Philipsen B, Deliens L (2011). Use of palliative care services and general practitioner visits at the end of life in The Netherlands and Belgium. J Pain Symptom Manage.

[21] Van den Block L, Van Casteren V, Deschepper R, Bossuyt N, Drieskens K, Bauwens S, Bilsen J, Deliens L (2007). Nationwide monitoring of end-of-life care via the Sentinel Network of General Practitioners in Belgium: the research protocol of the SENTI-MELC study. BMC Palliat Care.

[22] Meeussen K, Van den Block L, Echteld MA, Boffin N, Bilsen J, Van Casteren V, Abarshi E, Donker G, Onwuteaka-Philipsen B, Deliens L (2011). End-of-life care and circumstances of death in patients dying as a result of cancer in Belgium and the Netherlands: a retrospective comparative study. J Clin Oncol.

[23] Vega Alonso AT, Zurriaga Llorens O, Galmes Truyols A, Lozano Alonso JE, Paisan Maestro L, Gil Costa M, Herrero Llorente A, Ramos Aceitero JM (2006). Guide to the principles and methods of health sentinel networks in Spain. Gac Sanit.

[24] Deckers JGM, Paget WJ, Schellevis FG, Fleming DM (2006). European primary care surveillance networks: their structure and operation. Fam Pract.

[25] Donker GA: *Continuous Morbidity Registration Dutch Sentinel General Practice Network 2010. Annual report*. [http://www.nivel.nl/peilstations]

[26] Boffin N, Moreels S, Van Casteren V (2013). The Belgian Network of Sentinel General Practices Between 2007 and 2012: A Short Report.

[27] IIS (2009). Atención a Los Cuidados Paliativos: Organización en Las Comunidades Autónomas.

[CR28] The pre-publication history for this paper can be accessed here: http://www.biomedcentral.com/1472-684X/13/54/prepub

